# The bleeding weather: association of environmental changes with intracranial aneurysms’ rupture

**DOI:** 10.1007/s00701-025-06494-8

**Published:** 2025-03-22

**Authors:** Konstantinos Faropoulos, Georgios Kyriakou, Athina Kafritsa, Angeliki Papavasilopoulou, Andreas Grzeczinski, Artemios Artemiadis

**Affiliations:** 1 Department of Neurosurgery, Nicosia General Hospital (SHSO), 2029 Nicosia, Cyprus; 2 Medical School, University of Nicosia, Nicosia, Cyprus; 3 School of Medicine, University of Patras, Patras, Greece; 4 Medical School, European University of Cyprus, Nicosia, Cyprus; 5 Medical School, University of Cyprus, 1678 Nicosia, Cyprus; 6 Department of Neurology, Medical School, University of Cyprus, 1678 Nicosia, Cyprus; 7 Department of Neurology, Nicosia General Hospital (SHSO), 2029 Nicosia, Cyprus

**Keywords:** Aneurysmal subarachnoid hemorrhage, Environmental parameters, Dust, Bleeding severity

## Abstract

**Background:**

Various studies have attempted to link aneurysmal subarachnoid hemorrhage (aSAH) with environmental factors, yielding inconsistent results.

**Methods:**

We retrospectively collected clinical and demographic data from all patients who presented to our hospital with aSAH between January 1st, 2015, and December 31st, 2021.

**Results:**

Our study found a significant variation in the occurrence of aneurysm ruptures based on the day of the week, with more ruptures occurring on Mondays compared to Fridays. Additionally, we discovered that the amount of dust in the air prior to the ictus influenced the severity of the aSAH. In contrast, other environmental parameters such as atmospheric pressure, temperature, humidity, rain, wind speed, and cloud cover did not significantly impact the prevalence or severity of aSAH.

**Conclusions:**

To our knowledge, this is the first study to associate the severity of aSAH with atmospheric dust levels. Another key finding is the increased incidence of aneurysm ruptures on Mondays compared to Fridays.

## Introduction

A brain aneurysm occurs when a weakened blood vessel in the brain becomes abnormally enlarged, making it prone to rupture. Brain aneurysm incidence among adults is 3.2%, with a 1.6 times higher incidence in women [16]. While most aneurysms (50–80%) remain small and do not rupture, those that do lead to bleeding in the subarachnoid space, resulting in an aneurysmal subarachnoid hemorrhage (aSAH). The annual risk of brain aneurysm rupture ranges from 2 to 10% [16]. A ruptured brain aneurysm is a critical medical emergency with high rates of mortality and morbidity. About 40% of ruptures are fatal, and among survivors, up to 76% experience significant neurological deficits [16].

The severity and the potential complications of an aSAH have driven the neurosurgical community into an effort to create an algorithm that could predict or even prevent a devastating event like an aneurysm’s rupture. Inspired by the observation that there is a bi-annual peak of aSAH, some hypothesize that certain environmental factors could trigger or at least facilitate the rupture of an aneurysm. To support that hypothesis, many retrospective studies have been conducted over the years in different parts of the world. Notwithstanding, there is a discrepancy between these studies. A significant seasonality in the incidence of aSAH has been observed in many articles [1, 3–6, 9, 10, 13, 14], still, their findings are not unanimous and in some cases are conflicting. On one hand, subarachnoid hemorrhage risk is highest during spring and autumn according to some studies [4, 13, 14], during autumn and winter according to Law et al. [10], during October, December and January according to Ishihara [6], or during the spring [1]. Illy et al. [5] observed seasonality in the incidence of SAH, ICH and chronic subdural hematomas (CSDH), although this was not of statistical significance. On the other hand, Neidert et al. [12], Hakan et al. [2] and Shinkawa et al. [15] found no seasonal fluctuation of aSAH for patients with a subarachnoid hemorrhage that were analyzed. (even though the first was a single-center study and the latter was a study that evaluated aSAH and stroke patients). In the study by Miranpuri et al. [11], a morning peak for aSAH is demonstrated, while Patrice et al. [13] found that subarachnoid hemorrhage risk is lowest between midnight and 6:00 a.m.

Given the elusive nature of aSAH seasonality, researchers have shifted their focus to specific meteorological parameters, or combinations thereof, to better understand potential triggers. In detail, the incidence of aSAH tended to be increased with changes in barometric pressure [5, 8, 10, 13]. In other protocols, humidity [4] or cold and excessive atmospheric pressure are triggers for the occurrence of aSAH [3].

In contrast, various weather variables were shown to be without influence on aneurysm rupture [1, 12, 17]. However, these conclusions should be carefully interpreted, as the first is a huge meta-analysis, but the majority of the patients that included were stroke patients and only a few SAH patients, while the latter is a single-center study.

From the data above, it is clear that there is no solid conclusion, but only some indications. Additionally, the majority of the studies were performed in central and northern countries of the Northern Hemisphere and only a few in countries of the Southern Hemisphere. Cyprus is a European country located in southeast Europe near the Middle East and has several meteorological singularities that render it of particular research interest.

The aim of this study is to explore for the first time the association of meteorological conditions in Cyprus with aSAH.

## Materials and methods

In our study we retrospectively collected clinical and demographic data from all patients who presented to our National referral center between January 1st, 2015, and December 31st, 2021, with aSAH. Routine investigation of spontaneous aSAH in our Neurosurgery Department is performed with Computed Tomography Angiography (CTA), while Digital Subtraction Angiography (DSA) is reserved for cases with negative or inconclusive CTAs. For the data retrieval, ICD-10 codes for SAH, ruptured aneurysm and AVM (arteriovenous malformation) were used. All the patients with a SAΗ in the CT scan after a ruptured aneurysm, a ruptured aneurysm associated with an AVM or ‘benign’ perimesencephalic SAH with a negative DSA were included in our study. We excluded patients with traumatic SAH, tumour bleeding, AVM rupture with no associated aneurysm, coagulopathy/anticoagulant-induced SAH and spontaneous non-aneurysmal SAH.

Two independent physicians have calculated the Hunt and Hess scale and the modified Fischer score, based on the clinical status of the patients and the radiological findings.

The Hunt and Hess scale was: Grade 1 asymptomatic or minimal headache and slight neck stiffness, Grade 2 moderate to severe headache; neck stiffness; no neurologic deficit except cranial nerve palsy, Grade 3 drowsy; minimal neurologic deficit, Grade 4 stuporous; moderate to severe hemiparesis; possibly early decerebrate rigidity and vegetative disturbances, Grade 5 deep coma; decerebrate rigidity; moribund.

The Modified Fisher CT rating scale was: Grade 1 minimal or diffuse thin SAH without IVH, Grade 2 minimal or thin SAH with IVH, Grade 3 thick cisternal clot without IVH, Grade 4 cisternal clot with IVH.

Meteorological data were collected by the Department of meteorology, Ministry of Agriculture and Environment, in the weather stations of Larnaca Airport and Nicosia City. Data on air pollution were provided by the Air Quality Section of the Department of Labour Inspection of the Ministry of Labour and Social Insurance of the Government of Cyprus.

Weather conditions included: mean barometric pressure (hPa), mean temperature (°C), mean relative humidity (%), mean wind speed (m/s), mean cloud amount (/8) and four parameters of dust particles of 2.5 μm and 10 μm measured at traffic and residential areas. Weather parameters’ 1-day changes (i.e. weather parameter in the day of the event minus this parameter in the previous day and 2 days before (as further analysis) were also included in the analyses. Rainfall in Cyprus is limited, with only 63 days (2.5%) of moderate/heavy rain and 582 days (22.8%) of brief light rain during the study period. As a result, rain was excluded from all analyses.

### Statistical analysis

Descriptive statistics were used to present basic demographic data. The daily, monthly, seasonal variation (i.e. winter: December to February, spring: March to May, summer: June to August and autumn: September to November) and annual variation were analyzed by using goodness-of-fit chi-square test. To detect the effect of weather conditions on the risk of aSAH incidence we used the following statistical strategies: a. Univariate comparisons by using t-tests were done to find weather differences between eventful and non-eventful days, b. A negative binomial regression model was used with aSAH incidence as the dependent variable and weather parameters as predictors and c. All weather parameters were standardized as z-scores and were classified by using hierarchical cluster analysis based on the Ward method and squared Euclidean distance. This approach resulted in two clusters. Next chi-square test was used to explore if there was a difference in the number of eventful vs. uneventful days. Hierarchical cluster analysis for 1-day weather parameters changes could not identify discrete clusters for further study.

The level of significance was set at 0.05. Statistical analyses were made using IBM SPSS Statistics for Windows, Version 21.0. Armonk, NY: IBM Corp.

## Results

A total of 158 aSAH cases were found between January 1^st^, 2015, and December 31^st^, 2021. There were 154 eventful days (i.e. in 4 days two aSAH events occurred) and 2403 uneventful days. There were 101 (63.9%) females and 57 (36.1%) males with aSAH. Their mean age was 55.3 ± 13.6 years old (range 23 −105 years old). The commonest location of aneurysmal rupture in our series was at the site of AcomA, accounting for 57 patients or 36.1% of cases. The MCA region was the second most common site, and it included 47 patients or 29.8% of the cases Table 1.

**Table 1:** Demographic and clinical characteristics of the study sample (*N* = 158)

Characteristic	Value
Mean age (SD)	55.3 (13.6)
Females *N* (%)	101 (63.8)
Median mFS (IQR)	3 (2–4)
Median HHS (IQR)	3 (2–4)
Location *N* (%)	
* Anterior cerebral artery*	12 (7.6)
* Middle cerebral artery*	47 (29.85)
* Posterior cerebral artery*	3 (1.9)
* Internal carotid artery*	15 (9.5)
* Posterior inferior cerebellar artery*	4 (2.5)
* Superior cerebellar artery*	1 (0.6)
* Basilar artery*	10 (6.3)
* Vertebral artery*	1 (0.6)
* Anterior communicating artery*	57 (36.1)
* Posterior communicating artery*	8 (5.1)
Treatment	
* No treatment*	17 (10.8)
* Clipping*	21 (13.3)
* Embolism*	115 (72.8)
* Craniectomy*	5 (3.2)

*HHS* Hunt and Hess scale, *MFS* modified Fischer score

### Daily, monthly, seasonal and annual variation

The peak day incidence was on Monday (37/158, 23.4%) and the trough was on Friday (12/158, 7.6%). A significant day variation was found (*p* = 0.022). The peak monthly incidence was in January (18/158, 11.4%) and the though monthly incidence was in August (7/158, 4.4%). No significant monthly variation was found (*p* = 0.684). The peak seasonal incidence was in winter (48/158, 30.4%) and the trough was in summer (28/158, 17.7%). No significant monthly variation was found (*p* = 0.139) Table 2. The peak annual incidence was in 2019 (36/158, 22.8%) and the lowest in 2015 (5/158, 3.2%). A decrease in aSAH frequency was observed during the first two years of the COVID-19 pandemic, thus we are planning to compare the pre and post-pandemic era for possible association between COVID infection and an aSAH in an upcoming study. The annual variation from 2015 to 2021 was found significant (*p* < 0.001).

**Table 2:** Frequency (*N*, %) of aSAH cases by day, month and year

Monday	37 (23.4)	January	19 (12.2)	Winter	49 (31)	2015	5 (3.2)
Tuesday	18 (11.4)	February	15 (9.5)	Spring	39 (24.7)	2016	26 (16.5)
Wednesday	25 (15.8)	March	12 (7.6)	Summer	28 (17.7)	2017	31 (19.6)
Thursday	23 (14.6)	April	15 (9.5)	Autumn	42 (26.6)	2018	22 (13.9)
Friday	12 (7.6)	May	12 (7.6)			2019	36 (22.8)
Saturday	23 (14.6)	June	13 (8.2)			2020	27 (17.1)
Sunday	20 (12.7)	July	8 (5.1)			2021	11 (7)
		August	7 (4.4)				
		September	16 (10.1)				
		October	13 (8.2)				
		November	13 (8.2)				
		December	15 (9.5)				

### Univariate test of weather parameters

The differences of weather parameters between eventful and uneventful days are presented in Table 3. No statistically significant difference was found between eventful and uneventful days either with regards to weather parameters or to their 1-day changes.

**Table 3:** Differences in weather parameters between aSAH eventful and uneventful days

	Eventful days (*N* = 154)	Uneventful days (*N* = 2403)	Sig
Barometric Pressure (hPa)	1013.5 ± 5.3	1012.8 ± 5.9	0.158
D- Barometric Pressure (hPa)	0 ± 3.5	0 ± 3.3	0.895
Temperature (°C)	22.5 ± 7.8	23.5 ± 8.4	0.149
D- Temperature (°C)	0.2 ± 1.9	0 ± 1.9	0.179
Relative humidity (%)	51.8 ± 17.5	51.1 ± 17.1	0.618
D- Relative humidity (%)	−0.5 ± 11.0	0 ± 10.9	0.588
Wind speed (m/s)	3.7 ± 1.8	3.8 ± 1.9	0.529
D- Wind speed (m/s)	−0.1 ± 2.5	0 ± 2.3	0.689
Cloud amount (/8)	3.2 ± 2.5	2.9 ± 2.4	0.239
D- Cloud amount (/8)	0.1 ± 2.6	0 ± 2.5	0.588
Dust 2.5 μm traffic	16.9 ± 6.8	17.3 ± 10.5	0.719
D- Dust 2.5 μm traffic	0.3 ± 5.6	−0.1 ± 8.9	0.687
Dust 2.5 μm residential	14.2 ± 5.9	14.9 ± 9.9	0.377
D- Dust 2.5 μm residential	0.1 ± 5.9	0 ± 9.5	0.864
Dust 10 μm traffic	41.3 ± 24.9	41.8 ± 33.8	0.843
D- Dust 10 μm traffic	−0.9 ± 31.1	0.1 ± 30.1	0.726
Dust 10 μm residential	33.6 ± 23.8	34.7 ± 33.3	0.689
D- Dust 10 μm residential	−2.9 ± 35.6	0.1 ± 32.1	0.294

D-: difference between eventful and the previous day

Values represent means ± SD,

Student’s t-tests, *p* ≤ 0.05

### Negative binomial regression models

The negative binomial regression model for predicting aSAH after including all weather parameters as predictors was non-significant (F(9) = 8.014, *p* = 0.533) and none of the predictors was found significant. When 1-day weather changes were used as predictors, a statistically significant effect was found for the change of dust particles of 10 μm within the residential regions (β = −0.025, incidence rate ratio 0.98, 95% CI 0.953 – 0.999, *p* = 0.043) meaning that reduction of these particles in the air increased the chance of aSAH. However, it should be noted that the overall model was found to be non-significant (F(9) = 9.497, *p* = 0.393), so this finding needs caution. Lack of significance was also noted when 2-day changes were entered as predictors.

### Cluster analysis

Hierarchical cluster analysis resulted in two clusters (Table 4). The two clusters were significantly different in all weather conditions (data not shown). For the sake of interpretation, weather parameters were characterized as low and high. More specifically cluster 1 was characterized by high barometric pressure, low temperature, high humidity, low wind speed, high clouds and air dust concentrations. The opposite weather conditions were prevalent in cluster 2. Although in cluster one the aSAH eventful days were more frequent than in cluster 2, the difference was not significant (*p* = 0.445).

**Table 4:** Number of aSAH eventful and uneventful days within each weather cluster

	Eventful days	Uneventful days
Weather Cluster 1	72 (6.3%)	1070 (93.7%)
Weather Cluster 2	53 (5.4%)	924 (94.6%)

### Effect of on the severity of SAH

A secondary analysis of the role of weather and dust conditions on the SAH severity as ascertained by the Fisher scale was performed. The change of dust traffic 2.5 μm particles concentration between the day of the event and two days before was found to significantly affect Fisher score. More specifically, the mean concentration difference for Fisher scale equal to 1 was −2.9 ± 5.8 vs. 1.5 ± 5.8 for Fisher scale equal to 4 (*p* = 0.011). This means that lowering of these dust particles during the past two days was associated with less severity of the SAH event. All other weather or dust conditions did not affect SAH severity (data not shown).

### Treatment

The aneurysms were secured endovascularly in 115 patients, while in 21 cases surgical clipping was utilized. 5 patients had severe ischemia upon their arrival, thus only decompressive craniectomy was performed, while the clinical status of 17 patients was so poor that no surgical treatment was offered Table 1.

## Discussion

There are several well-known factors like hypertension, smoking or collagen deficiency syndromes and others that contribute to the rupture of an aneurysm. On the other hand, it is not clear if environmental variables like temperature, atmospheric pressure, and humidity or air pollution can cause the rupture of an aneurysm. So far, numerous studies have been conducted, trying to correlate the rupture of aneurysms with seasonal and climatic conditions. Most of these studies were performed in central or northern countries of the Northern Hemisphere and a few in countries of the Southern Hemisphere.

To our knowledge, no study that takes into account the local environmental parameters of our country or of countries worldwide with similar environmental parameters has been published. Having that in mind, we conducted a retrospective study seeking for environmental conditions that could increase the rate of aSAH in Cyprus. Cyprus is an island in the Northeast Mediterranean Sea, near the coast of the Middle East. As a result, the country has some unique meteorological characteristics. In general, the weather is hot, and the humidity is high, especially during summer, there is increased sunshine for at least 8 months, while the climate is significantly affected by warm waves of heat and dust from Africa and Middle East. Parameters like atmospheric pressure, temperature, humidity, rain, wind speed, clouds and the amount of dust on the day of the rupture, were tried to be associated with aSAH prevalence and severity.

The peak seasonal incidence was in winter, and the trough was in summer. In the same context, the prevalence of aSAH was increased in January and reduced in August. This is in accordance with the Law et al. [10] study that also described a seasonal peak of aSAH in winter (December to February), a seasonal low in summer and a monthly peak in January in a series of 135 patients that took place in Hong Kong. Similarly, Ishihara [6] and his team observed that the peak onset of SAH was in December and January in a large series of 5007 patients in Japan. On the other hand, various studies found seasonality in different seasons of the year [4, 13, 14] and other studies failed to find any statistically significant seasonality [2, 7, 12, 15]. The discrepancy between all the studies perhaps is the result of differences in their methodology. Additionally, given that there is no consensus among neurosurgeons worldwide about if there is seasonality in the rupture of aneurysms, perhaps local, unique environmental variations cause the rupture of an aneurysm and these variations are not the same all over the world.

In our study, a significant day variation was found, as the peak day incidence for aSAH was on Monday and the trough was on Friday. The latter finding is statistically important and to our knowledge, it is the first time that a specific day has been associated with the danger of an aneurysm’s rupture. Interestingly, an increased occurrence of myocardial infarctions was noted on Mondays [18] and it was hypothetized that the stress in the working population on the first work day of the week could trigger the MI. Even though the same mechanism could facilitate the rupture on an aneurysm, uture studies on the day-variability of aSAH will shed more light on this subject.

The comparison of the changes of weather parameters between eventful (aSAH) and uneventful (no aSAH) days found no statistically significant difference between eventful and uneventful days either with regards to weather parameters or to their 1-day changes. A negative binomial regression model and a Cluster analysis were performed looking for a missing significant relationship. However, no significance was found. Our results are similar to the findings of previous studies, where the relation of aSAH with environmental parameters was not statistically significant [1, 12, 17].

Just like us, Neidert et al. [12] tried to correlate environmental variables with clinical data of the patients with an aSAH like initial GCS, H + H and Fisher score, but no significant correlation was found.

The secondary analysis on the role of weather and dust conditions on the aSAH severity as ascertained by the Fisher scale revealed that a lowering of 2.5 μm traffic dust particles during the past two days from the ictus was associated with less severity of the SAH event.To our knowledge, this is the first instance in the literature linking atmospheric dust levels to aSAH severity as measured by the modified Fisher scale, which is also associated with a higher risk of severe vasospasm. The underlying pathophysiological mechanisms remain unclear, but respiratory discomfort caused by poor air quality from elevated dust levels before the event may contribute to increased intracranial pressure, and that could lead to a more devastating rupture of the aneurysm and increased blood delivery in the subarachnoid space. This topic will be addressed in an upcoming study. Finally, we should acknowledge some limitations of our study. It is a single-center study, but, considering that our hospital is the neurosurgery referral center, virtually all the country’s cases of ruptured aneurysms are transferred here. The number of patients is not that large, but we must keep in mind that we live in a small country. To make the data analysis feasible, we have considered that the environmental measurements that were taken from two stations (Larnaca airport and Nicosia station) can be applied to the entire island. That assumption, even if it looks oversimplified, should not be considered wrong, as Cyprus is a small country, with no significant differences in its climate within its different provinces.

## Conclusion

Currently, there is no consensus among the neurosurgical community if there are environmental variables that could trigger an aSAH. To our knowledge, our study is the first to correlate the severity of the aSAH with the amount of dust in the air on the island of Cyprus the days before the ictus. A second sub-result of our study is that significantly more aneurysms were found to rupture on Monday than on Friday. Our findings should be replicated by future studies.

## Data Availability

No datasets were generated or analysed during the current study.

## Abbreviations

IVH: Intra-ventricular hemorrhage
aSAH: Aneurysmal subarachnoid hemorrhage
CSDH: Chronic subdural hematomas
